# Reconfigurable free-space mode generation and detection enabled by an active photonic integrated circuit coupled to a passive mode-selective interface

**DOI:** 10.1038/s42005-026-02522-w

**Published:** 2026-03-05

**Authors:** Aleksandr Boldin, Ultan J. Daly, Maziyar Milanizadeh, Rakan Alsaigh, Zhaozhong Chen, David A. B. Miller, Francesco Morichetti, Martin P. J. Lavery

**Affiliations:** 1 James Watt School of Engineering, University of Glasgow, Glasgow, UK; 2 Dipartimento di Elettronica, Informazione e Bioingegneria, Politecnico di Milano, Milan, Italy; 3 Ginzton Laboratory, Stanford University, Stanford, CA USA; 4 Present Address: Quantum Technologies, Toronto, ON Canada

**Keywords:** Integrated optics, Adaptive optics, Transformation optics, Fibre optics and optical communications

## Abstract

Optical mode-sorting and generation are used for a wide range of quantum, sensing and communication applications. High-speed switching between mode sets would allow photonics systems to react to changes in propagation channel in real time. Photonic-Integrated-Circuits (PICs) with phased arrays can rapidly reconfigure their function at MHz depending on installed modulators. As a reconfigurable photonic system, PIC can reconfigure significantly fast than spatial light modulators that are limited to less than 1 kHz. However, phased-arrays are bound by two-dimensional Nyquist-sampling limit and spacing between array elements leads to grating lobe formation, where many additional array elements are needed to mitigate these effects. We leverage a passive Multiple-Plane-Light-Converters (MPLC) as an Optical Mode-Selective Interface (OMSI), coupling a basis set of free-space Hermite Gaussian (HG) modes directly into an active optical mesh implemented on a PIC. The active mesh can generate or sort any free-space mode-set that can be created by a linear superposition of 15 HG modes. Without changing the physical system, we demonstrate the generation and sorting of four orthogonal mode groups, each with 15 modes, achieving a mean intermodal crosstalk for sorting of -22 dB. This approach can allow for rapidly reconfigurable mode-sorters that could be used for quantum or classical communications or sensing applications.

## Abstract (web summary)

Rapid switching between optical mode sets is critical for adaptive photonic systems, yet spatial light modulators are limited to sub-kilohertz speeds. The authors devise a reconfigurable mode-sorter by combining a passive multi-plane light converter with an active photonic integrated circuit, able to generate four orhogonal mode groups with -22 dB crosstalk for sorting.

## Introduction

PICs are a rapidly maturing research area, with applications across communications, quantum optics, sensing, biomedical devices and optical computing^1–5^. These chips can be used for rapidly controlling the path that optical signals take through planar waveguides and complex optical systems, such as Mach–Zehnder interferometers (MZI)^6, 7^. Compact and relatively phase-stable on-chip platforms can readily allow for the realization of multiple cascaded interferometers on chip, which would not be viable in benchtop optical systems^3^. This has been a key driver for research in use of PICs for applications in optical switching^8^, quantum computing^9^, neuromorphic computing^10^, and optical modulation^11^. PICs have been widely used for many applications within fiber optical systems due to the availability of efficient approaches for direct coupling, such as edge or grating couplers^12–14^. Recently, the rapid re-configurability of PIC systems has led to increased interest in utilizing PIC for free-space optical (FSO) applications such as LiDAR^15^, re-configurable optics^16^ and line-of-sight communications^1^. This presents an opportunity for PIC technologies to form the basis of future mode-sorting systems that can rapidly change the mode-group being sorted. Such systems could be used widely for adaptive optical systems that are required to tune the orthogonal modes to match a particular channel, such as quantum communication based on eigen modes, or optical sensing with tailored spatial fields.

Optical systems design for mode-sorting has been an area of interest for more than 30 years, during which a range of technologies has been proposed to perform all optical transformations of light; such that the power associated with a specific free-space propagating optical mode, such as a Laguerre Gaussian (LG) or HG optical modes, is coupled efficiently into a single output channel or detector. Conventionally, mode sorters are passive optical elements designed for a particular fixed set of optical modes, based on specific geometric transformations^17–20^. An exciting method for optical transformation is the use of MPLC approaches. These can be either in the form of a known geometrical transformation, such as a log-polar transformation for sorting Orbital Angular Momentum (OAM) modes^19^, or inverse design methods^21, 22^ for sorting HG or LG modes^20^. The number of phase screens, *N*_*p*_, in an MPLC required for arbitrary mode-sorting is generally considered as *N*_*p*_ = (2*N*_*m*_ + 1), where *N*_*m*_ is the number of modes being sorted^23^. Reducing the number of screens used will generally result in additional crosstalk between modes, where the specific acceptable tolerance is application-specific. As demonstrated by Fontaine et al., for HG mode sorting suitable for communication systems, six planes can be used due to geometric symmetries between the input mode and a triangular output spot array for more than 210 modes^20^. MPLCs have been demonstrated to perform a wide variety of unitary operations at high quality by using only a few phase modulation planes^24^. Optimized inverse design methods can be used to improve the performance of MPLCs, such as gradient ascent-based methods^25^. However, these approaches cannot be used for a mode set that is different from the one for which the optical elements are designed. Reconfigurable approaches based on spatial light modulators (SLM) have been demonstrated, where the through numerical computation or experimental variation in the of phase screens of the MPLC is updated to match a particular mode basis that can be transformed by the maximum number of phase screens that can be displayed on an SLM. For true arbitrary mode sorting for applications such as beam-steering or real-time adaptive mitigation of atmospherically-induced distortions^26–28^; the number of required screens would need to equal the number of modes being sorted which is often difficult to implement on SLMs with limited aperture size^23^. The highest number we are aware of being demonstrated on an SLM is ten planes^29^, which limits the transformation achievable with this fixed number of MPLC planes^23^. If a fixed mode set can be used, such as HG modes, a highly optimized and large bandwidth MPLC could be used to sort 100s of modes^20^. Combining a highly efficient MPLC with a PIC has been suggested as a method to enable coherent beam combining for communication downlink terminals^30^. Therefore, an optical system that comprises a highly optimized fixed MPLC, with a reconfigurable PIC, could provide a flexible and efficient platform for general-purpose mode generation and measurement.

PIC technologies offer a photonic platform that can simultaneously control the phase and intensity of light at many MHz or GHz^31^, making it an ideal optical control system for these applications. It is common for planar waveguides to be single-mode; therefore, to couple into free space, grating couplers are commonly used. However, a core challenge for any phased array antenna, whether in acoustics, radio, or photonics, is a requirement for the array elements to be placed half a wavelength apart to prevent the generation of grating lobes that limit coupling efficiency and field quality of generated spatial fields^32–34^. Approaches to mitigate this fill-factor issue for LIDAR applications have been proposed through a combination of closely packed linear arrays for 1D steering with wavelength tuning to provide 2D beam steering^35^. However, continuous tuning of wavelength can limit the applicability of this approach where multiple or specific wavelengths are required, such as for wavelength-division multiplexing (WDM) in optical communication^36^. Inverse-designed couplers have shown promise for direct generation of fixed specific modes using 2D patterned couplers^37^. However, a technology that leverages the efficiency of MPLCs and reconfiguration of PICs could be revolutionary for FSO for the rapid generation and sorting of arbitrary optical fields.

**Fig. 1:**
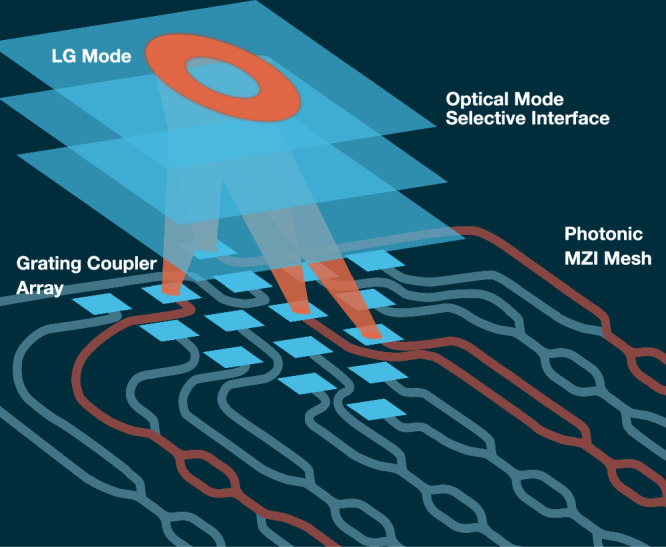
Concept. A mode-selective optical interface passively decomposes the optical input into a triangular array of spots collected by grating couplers (blue squared elements). In the figure, we depict an Laguerre Gaussian (LG) mode with *ℓ* = 2 being coupled into multiple waveguides. This arrangement mitigates losses due to low fill factor of the array and reduces the number of required Mach–Zehnder interferometers (MZI) on chip.

In this article, we demonstrate a reconfigurable mode-sorter that implements an MPLC as an OMSI for a PIC comprising a cascaded binary-tree mesh of MZIs, Fig. 1. By leveraging the inherit rapid reconfigurability of the PIC platform, we can generate and detect arbitrary spatial modes within *N*_*m*_-dimensional Hilbert space defined by the OMSI. One complete basis for this Hilbert space is the set of spatial wave functions that map one-by-one to specific on-chip couplers. To optimize power coupling into each on-chip coupler, we experimentally recorded the complex spatial profile of the transmitted mode from each coupler. These modes were subsequently used to compute the MPLC phase masks, allowing them to minimize mode-dependent loss by approximately 3 dB. This allows us to minimize the number of planes required to 6, as the transformation geometry is similar to previous MPLC systems^20^. Each coupler will act as either an optical input (I) or output (O), for mode sorting or mode generation, respectively. Therefore, each coupler is considered as an input-output channel (IO). The MZIs can then be software reconfigured to transform to any convenient mode basis in the OMSI-defined Hilbert space, with no change in the physical optics used or OMSI phase masks. We utilize a binary tree mesh for our demonstration, but note that the approach could be readily combined with fully connected multiple-input and multiple-output PIC-based MZI networks. Our results show that this combined MPLC and PIC approach can be used to sort or generate arbitrary modes compatible with free-space channels. Our re-configurable system can be tailored to sort or generate any orthogonal set of spatial modes or arbitrary superpositions of modes. We have successfully sorted and generated spatial modes in any of the LG or HG bases, as well as arbitrary modes in the same Hilbert space, with a mean value of inter-mode crosstalk (XT) for sorting of −22 dB without any change to the physical optical system. We measure that our system can be reconfigured faster than 19.12 kHz. This is limited by: (1) the time for the electrical current to induce heat-driven expansion in the waveguide that results in a 2*π* additional path-length, and (2) the time required for the modulator to cool back to ambient once the electrical current is removed, where the waveguide returns to its original length. Our system leverages the fundamental principle of the orthogonality of optical spatial modes to resolve the fill-factor issue, which means the OMSI can reduce the number of optical-IO and control elements required for field shaping or mode-sorting by more than a factor of 4, assuming a square array of optical-IO detecting LG beams. This approach opens the possibility for realizing efficient PIC-based adaptive optics and for the high-speed creation of channel-defined spatial modes for communication or sensing applications in complex environments. More generally, incorporating an OMSI in integrated systems could alleviate many of the issues related to scaling of PIC technologies, reducing both optical losses and complexity for future systems that utilize PIC devices.

## Results

### Concept

#### Optical mode-selective interface for integrated photonics

Our project utilizes an MPLC as a passive optical interface to mode-match from free-space to our PIC that is electrically controlled to change the specific mode set the system is sorting. This allows for rapid reconfiguration of the optical modes being sorted by the system. This MPLC, acting as an OMSI is similar in function to a mode-sorter, where it transforms HG modes into a series of near-Gaussian spots that are designed to efficiently couple into 15 on-chip grating couplers. Therefore, the OMSI eliminates the losses and grating lobes associated with pixelated phased-array transmitters or receivers. The OMSI defines a 15 dimensional mathematical (Hilbert) space. One complete basis for this Hilbert space is the set of input functions that map one-by-one to specific on-chip coupler. For example, the MPLC in our feasibility demonstration is designed to map 15 different HG modes in this way. The linear superposition of any fixed complete spatial mode set, such as HG, can be used to represent many unique orthogonal basis sets that are tailored to a given application. Therefore, by controlling the relative power and phase between each of these modes using a PIC, the system can subsequently generate or sort any spatial mode that can be formed as a linear superposition of the Hilbert space defined by a fixed mode set of the input coupler. The Nyquist sampling limit requires a minimum of two pixels or samples to recover a particular spatial frequency. For a 2D array to measure a 2D spatial complex amplitude profile, it would require at least twice the number of pixels as the number of spatial frequencies in each dimension. As the OMSI maps a particular mode with a 2D spatial complex amplitude profile into one optical input or output, the PIC itself is no longer required to meet the sample limits imposed by Nyquist, as the OMSI defines the spatial sampling limits.

**Fig. 2:**
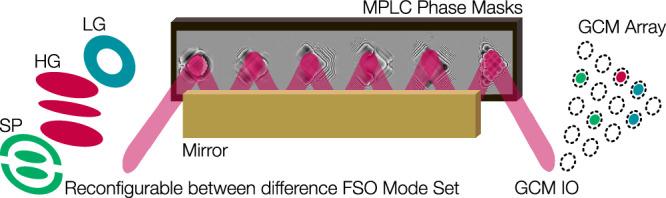
Optical mode-selective interface (OMSI). A multiple plane light converter (MPLC) can be used as a passive OMSI for efficient coupling into a reconfigurable photonics integrated circuits (PIC). This is composed of six phase masks displayed on a spatial light modulator in a folded optical path. Between each phase mask, the optical beam is reflected by a mirror to provide the required 28 mm propagation distance between surfaces. The MPLC is designed to transform each Hermite Gaussian (HG) mode into a grating coupler mode (GCM) and separate them such that each is incident on one particular position in the GCM array. Other mode groups can be sorted by using the beam to coherently combine multiple spots that are incident on multiple grating couplers for a range of different mode sets, including Laguerre Gaussian (LG) and superposition (SP) modes. The optical beam that is from the foundry’s standard grating couplers produce near-Gaussian, where each was measured individually to accurately mode-match to each coupler.

We utilize HG modes as our native basis set, as they form a complete set in Cartesian coordinates; this allows for sorting and generation of a wide variety of arbitrary modes that have 2D complex amplitude profiles. This is achieved through the linear superposition of the 15 HG modes, each with specifically selected relative intensity and phase. To design the OMSI, we use the inverse-design method that has been used widely for the creation of MPLC devices^20–22^. This method relies on an iterative back-propagation simulation to generate a series of optimized optical elements that will enable particular field transformations between two optical planes^21^. In our OMSI, we define our two planes as (i) FSO Modes Input/Output (IO) plane and (ii) experimentally measured grating coupler mode (GCM) from each coupler in a triangular array for both coupling into and out of the PIC, see Fig. 2. We refer to this plane as grating coupler mode input/output (GCM IO) array. In our implementation of the MPLC iterative algorithm^21^, we utilize forward and backward propagation simulations between the FSO modes IO plane and GCM planes to determine the required phase profile at six fixed mask positions to perform the required transformation, as shown in Fig. 2. The spacing between planes was determined to be 28 mm, by performing a computational optimization based on the input and out field distributions. The inter-plane distance is similar to that reported in literature^20^. During any iteration, the required transformation is determined for each OMSI mode individually, and the phase profiles for all modes are then superimposed. Therefore, as the MPLC is required to efficiently couple light into an array of grating couplers, as shown in Fig. 1, we directly measured the phase and intensity profile of light emitted from each coupler, acquiring grating coupler modes (GCM). Each GCM in the array is used with our iterative calculation to define the “GCA IO” plane. The required phase profile of each mask, ${\phi }_{p}(x,y)=\,{\mbox{arg}}\,\left[{M}_{p}(x,y)\right]$, is calculated using

1 $${M}_{p}(x,y)={\sum }_{i=1}^{\,{\mbox{N}}}{E}_{{{\mbox{F}}}_{p}}(x,y)\,{E}_{{{\mbox{B}}}_{p}}^{* }(x,y)$$

determining the overlap between the complex amplitude of the forward-propagating beam, ${E}_{{F}_{p}}(x,y)$, and the complex amplitude of the backward-propagating beam, ${E}_{{B}_{p}}(x,y)$, superimposed for N beams carrying sorted modes^20^. Through repeated iterations of propagation modeling using plane-wave decomposition, the phase profile of each mask is re-evaluated at each iteration, gradually converging towards an optimum solution. As plane-wave decomposition propagation modeling is based on Fourier transforms of the complex amplitude, Fourier filtering is critical to prevent wrapping effects for power that would physically be lost during any single propagation step. Therefore, we impose a limit on the range of spatial frequencies available to the simulation by truncating the transfer function of free space defined in spatial frequency coordinates with a binary circular aperture. The smooth profile of each mask indicates that this effective filtering has been achieved, see Fig. 2.

#### Photonic integrated circuit

**Fig. 3:**
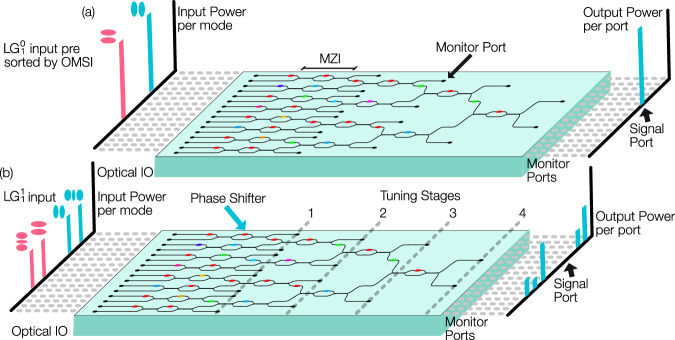
Photonic mesh. The PIC used in this feasibility study utilizes a 16 by 1 binary-tree structure, which comprises 16 Optical inputs and outputs (IO), 15 monitor channels and one signal port. We utilized 15 Optical-IO for the optical field from Optical mode-selective interface (OMSI), whereas the 16th channel was not used in our study. Horizontal dashed lines are a visual aid to indicate the associated bar in the power bar chart with the optical IO or monitors ports on the chip. **a** Mode sorting is achieved by tuning the Mach-Zehnder interferometers (MZI) mesh by minimizing the power in the monitor channels for a given input mode. This is done in 4 stages, where the near vertical dashed lines indicate output monitors used for each step of power minimization. **b** When a different optical mode is incident on the OMSI, and phase-shifter values are maintained for the mode in (**a**), no power is detected at the signal port and power is distributed to other monitor channels. In an N x N mesh, our monitors would be connected to additional MZIs.

Our PIC is a binary-tree mesh^38^ and represents a single self-configuring layer in what could be scaled to an N × N fully connected mesh of N such layers for multiple input and multiple output transformations^38, 39^. Such N × N can be readily manufactured at a foundry, where 4 × 4 and 6 × 6 have been demonstrated for optical computing^40, 41^, along with 8 × 8 have been demonstrated for quantum applications^41^. The engineering challenges in scaling integrated photonics are rapidly being resolved to support quantum computing applications^42^. With growing industrial interest in the use of PICs, the engineering challenges of developing large optical meshes are being rapidly resolved, such as the Google X Taara-chip for free-space optics will provide platforms for controlling 100s or 1000s of on-chip components. As these developments mature, it is important to investigate scalable coupling solutions. Therefore, as the performance of an N × N can be readily replicated in lower complexity devices, we chose to conduct our experiments with an N × 1 unit cell of what could become an N × N in the near future, as the feasibility of the approach can be fully explored. Our PIC was fabricated using the standard 220 nm silicon photonics (SiP) platform at the AMF foundry. The design wavelength for the PIC is 1550 nm, therefore, 500 nm-wide rectangular section waveguides are utilized. The chip has 16 optical IOs, with 4 layers of MZIs to control the mixing of the optical fields from each input mode, Fig. 3a. Each MZI consists of two 3 dB evanescent waveguide splitters that are formed from two waveguides 300 nm apart, with an interaction length of 40 μm. Each 3 dB splitter supports two input and output branches. To control the power and phase of the light after propagation through each MZI, a thermal phase-shifter is placed before each splitter. One of the two outputs from the splitter is routed into the next layer of the mesh. The other output branch is connected to an output monitor, where the power is measured during PIC tuning, Fig. 3b. Control of each phase-shifter is achieved by applying electrical current to a titanium nitride (TiN) strip 2 μm wide by 80 μm long, which slightly heats the silicon waveguide, increasing the path length compared to the adjacent branch within the MZI. This arrangement can be tuned to direct the power from a particular input field to one signal port. As the free-space beam incident on the OMSI is decomposed into HG modes, the circuit can be adjusted to balance the power and relative phases by sequentially minimizing the power recorded at the monitor port connected to each MZI. This tuning is achieved by digitally varying the voltage at each phase-shifter and performing a Nelder–Mead optimization^43^ algorithm in real time. The circuit arrangement allows for simultaneous tuning of all MZIs within one layer, therefore, the full alignment can be achieved in four sequential steps. Our circuit is thermally bonded to an aluminum heat sink and a thermo-electric controller to prevent thermal cross-coupling to other MZIs and to maintain an average circuit temperature of 30 °C. Further, the algorithm allows for recorded heater voltages to be recalled at a later date for spatial mode sorting or generation of structured light from the circuit when combined with the OMSI. When a different input field, orthogonal to the field the circuit has been tuned for, is presented, the power will be distributed to other monitor ports, which can either be directly measured by the monitor ports to estimate expected crosstalk or connected to further cascaded meshes for N × N sorting applications. In our implementation, the power in each monitor port is measured by a short-wave infrared (SWIR) camera, and the heater voltages are automatically controlled through the use of a multichannel analog voltage source (National Instruments PCIe-6738).

For the generation of arbitrary spatial fields, we can free-space couple a single-mode optical source into the signal port of the mesh. Similarly to the process of sorting arbitrary modes, we can create a desired optical wavefront through the linear superposition of the OMSI’s supported modes. Through software, the voltage settings for each heater can be recalled to create the required phase and intensity at each optical-IO port to generate the desired optical output mode from the OMSI. Multiple approaches to controlling the output can be realized, including full calibration of the circuit through fitting to an algorithmic representation of the mesh to allow for the computational design of desired outputs^44^. Our focus is on the feasibility of the OMSI for the generation and detection of arbitrary spatial fields, where our measure-and-recall approach offers a simplified method to demonstrate system performance.

### Arbitrary optical mode sorting

We investigate the feasibility of using our OMSI and PIC system for the sequential sorting of a range of spatial modes. Our PIC architecture is a binary-tree mesh, which can be configured to couple light from the optical IOs to the signal port for a given mode and simultaneously reject all modes that are orthogonal to the mode being sorted. By using cascaded binary trees or other forms of M x N meshes, multiple modes within a particular mode set could be sorted simultaneously^7^. To determine the performance of our system, we utilize the experimental setup shown in the “Methods” section.

The PIC can be configured to sort any coherent input field; therefore, we chose to prime our system using test modes supplied by an external spatial light modulator. Our approach allows for simultaneous calibration of the free-space alignment of the SLM to the OMSI and the OMSI to the PIC. The PIC can be calibrated to enable numerical programming of arbitrary fields; however, our approach limits additional calibration errors within the setup to specifically test the functionality of the full optical system.

**Fig. 4:**
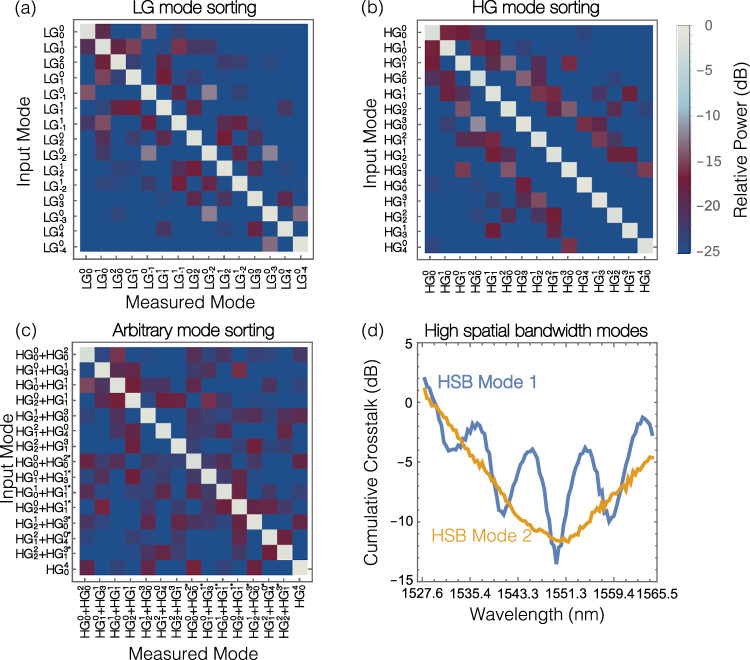
Mode sorting. Crosstalk matrices indicate the measured power coupled into each of the sorted channels. Without changing the physical system, **a** LG, **b** HG, and **c** SP modes are successfully sorted with low mean crosstalk at 1550 nm. The * term denotes that the modal component is *π* phase shifted with respect to the other mode in the superposition. **d** Changing wavelength without re-tuning the system will result in additional modal crosstalk and beating behavior for some modes.

We generate and sort 60 modes across four different modal basis sets: LG, HG, and two orthogonal arbitrary mode sets. The first arbitrary set is composed of linear superpositions (SP) of pairs of HG modes with specifically chosen relative phase shifts, as shown in Fig. 4. The second arbitrary set contains high spatial bandwidth (HSB) modes with orthogonal distributions of intensity and phase, where each mode results in power reaching each of the 15 optical IOs. We determine the required voltage settings by directly illuminating the system’s input with the desired optical field from an external SLM and tuning the mesh such that the power is combined at its signal port. The voltage settings are then stored for each mode. By tracking power in monitor channels using a camera-based control loop running at approximately 10 Hz, the mesh is fully tuned within 100–150 voltage adjustments. This tuning process can be performed several hours before crosstalk data is recorded, as the temperature stabilization of the PIC is maintained at 30 °C to prevent a change in the response of the thermal phase-shifters. To measure the crosstalk, we first tune the mesh to direct all the power to the signal port for a particular input mode, M_i_, then sequentially step through each of the 15 modes from the same orthogonal mode set, recording optical power coupled to the signal port. This process can be repeated by setting M_i_ sequentially to each mode in the mode set to record a 2-dimensional XT matrix.

Our results show the inter-modal XT measured for a set of different spatial modes and determine three key metrics that are presented in Table 1. These metrics are: (1) the mean measured XT power (the off-diagonal measurements in the XT matrix) as a ratio to the maximum on-diagonal measurement, *s*_mxt_; (2) the cumulative XT power, defined as the ratio of the total off-diagonal power and total on-diagonal power, *s*_cxt_; and (3) the estimated channel capacity when using the measured value of *s*_cxt_ and assuming electrical noise equivalent to a single channel SNR of 27 dB and bandwidth of 1 Hz. See Eqs (3) and (4) in Section “Crosstalk calculations” and Eq (2) in Section “Channel capacity” for details of the calculation method for each metric.

**Table 1:** XT performance for the four different orthogonal mode-sets experimentally investigated with an input wavelength of 1550 nm

Mode-set	LG	HG	SP	HSB
Mean per channel XT (*s*_mxt_ dB)	−22.1	−22.3	−22.0	−21.9
15-mode cumul. XT (*s*_cxt_ dB)	−10.11	−9.9	−9.86	−10.9
Est. Chan. Cap. (bits/S/Hz)	52.0	49.1	48.7	55.3

Estimated channel capacity is determined for 1550 nm, wavelength-dependent changes in SNR or XT could reduce the performance.

crosstalk arises from a few elements in the optical system. Firstly, our implementation uses two independent SLMs for the OMSI and the reference field generator, respectively. The Holoeye Pluto 2.1 SLM has a fill factor of 93 %, which subsequently generates additional diffracted orders that contribute to background noise. The propagation of the diffracted orders through the optical system from the reference generator is minimized by the use of a spatial filter. However, the SLM used for implementing the OMSI is operated in zero-order (as opposed to a blazed grating), therefore, 0.5 % back-reflection from each surface will lead to further contribution to the optical background. Additionally, the relative misalignment of each plane within the OMSI contributes to a slight disruption of orthogonality for the HG mode sorting achieved by the OMSI. We note that commercial devices which similarly use multiple planes have been demonstrated with low modal crosstalk of around −27 dB^45^, and that an MPLC for non-PIC-based demultiplexing built using SLMs has similar modal crosstalk performance to our implementation^20^. Our setup calibration has been shown to have stability over the course of days in an environmentally controlled laboratory setting. Packaged systems would be expected to maintain alignment for the longer term, and the PIC itself could be used to mitigate changes in the optical alignment of the OMSI.

Our PIC and OMSI are both wavelength-dependent; therefore, a change in the wavelength or usage of wavelength division multiplexing will change the crosstalk performance. We maintained our PIC tuning for the reference mode at 1550 nm while varying the input wavelength across the 92 C-band channels, from 1527.6 nm to 1565.5 nm. At each wavelength, the hologram on SLM1 was recalculated and the crosstalk matrix was remeasured. To determine the effect of changing wavelength, we consider the power ratio between the expected channel and the cumulative power in all other channels, as shown in Fig. 4. Fundamental sources of degradation in performance result from changes in the splitting ratio of the 3 dB waveguide splitters designed for 1550 nm, dispersion in the waveguides, and from the reduction in coupling due to the wavelength selectivity of the grating couplers. Although a change in crosstalk is measured, a broad bandwidth across the C-band still provides over 70 wavelength channels that have a cumulative crosstalk lower than 8 dB. These results strongly indicate that this approach could readily be used for hybrid space-and-wavelength division multiplexing communication schemes. We note as the spatial light modulator is pixelated, with 8 bit phase resolution, high-order aberrations are possible which could result in scattered light that could contribute to measured crosstalk. As this is a feasibility investigation, we expect that with future improvements, we could lower the intermodal crosstalk through the usage of a fabricated optical MPLC-based mode-sorter, such as that demonstrated in ref. ^45^.

### Arbitrary optical generation

Any arbitrary set in the same Hilbert space can be represented as a linear superposition of the native HG modes of the OMSI. To generate an arbitrary mode in this space, we use the PIC to control the relative phase and intensity at each optical IO port through voltage control of both heaters within each MZI. To determine the performance of our system, we utilize the experimental setup shown in Methods. We note that system calibration can also be used with a similar binary tree mesh to computationally design the output fields; however, the presented results are focused on the optical system performance and not control strategies.

**Fig. 5:**
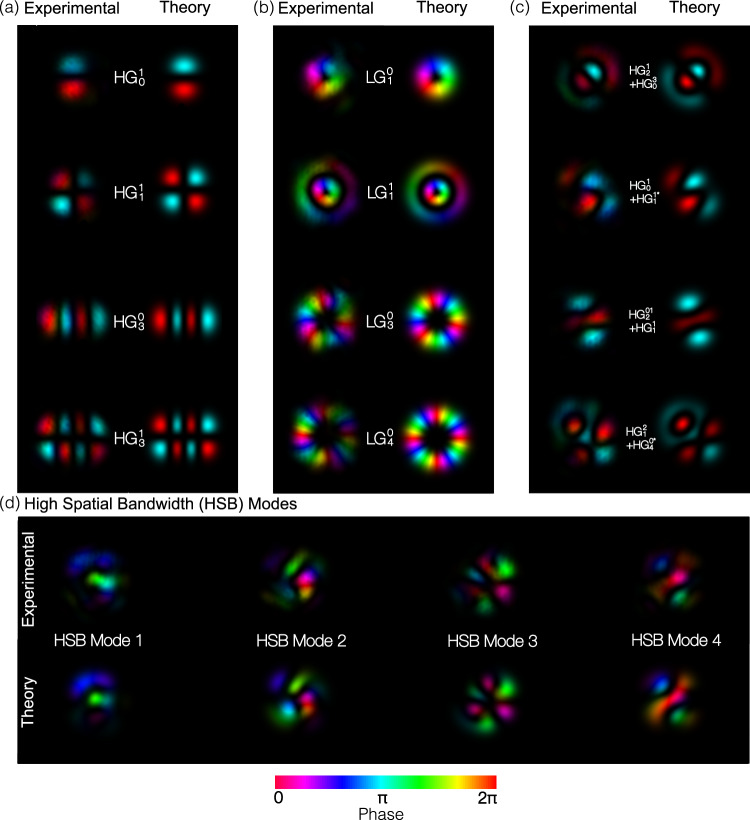
Mode generation. Using the combination of OMSI and PIC the generation of grating-lobe-free optical modes is demonstrated for **a** HG, **b** LG, **c** superposition modes and **d** high spatial bandwidth modes comprising an orthogonal set that are linear superpositions of all 15 base HG modes.

It can be seen in Fig. 5a that the generated optical fields do not have the diffraction patterns with multiple diffraction orders that commonly occur when using phased array antennas with grating couplers. To assess the performance of the system, off-axis digital holography is used to recover complex optical fields generated by the combination of PIC and OMSI, in “Methods”. Using the complex amplitudes of the illumination beams, the overlap integral can be calculated to determine the similarity of the generated optical field with the one that originally illuminated the chip for tuning. Our results indicate an overlap greater than 85% when compared to the expected theoretical fields for LG modes, where experimental limitations, such as scatter, can substantially lower the measured overlap percentage. To assess systematic errors in the illumination and measurement system, we have also directly measured the overlap integral for the illumination fields generated from the SLM to be approximately 94%. This indicates that our prototype system has 10% additional error with respect to the modes generated from the SLM. As our OMSI is free-space aligned, thermal or mechanical misalignment can occur, leading to errors both in the recording of the fields and subsequent generation. Further, as SLMs are reflective and pixelated, scattering effects can occur, resulting in the generation of a small amount of speckle that can substantially affect the measured overlap integral. Using a Fourier filter, we remove the small amount of power that is scattered in high-order spatial components from grating couplers and reflected orders from the SLM used to realize the OMSI. By measuring the power lost at the aperture, we estimate that 2 dB of the power is scattered by the optical system. These losses and errors would be substantially minimized through the fabrication of OMSI using diamond turning or lithography^19, 45^.

A core advantage of building a system on an integrated photonic platform is the potential speed of reconfigurability. We tested rapid switching between spatial modes and measured it at 19.12 kHz. Our results indicate that there is minimal degradation in the recorded optical fields arising from such fast modulation. The current implementation utilizes heaters, which need time to cool down when a high voltage is set. This relaxation time was measured to be approximately 66 μs. Utilizing other phase modulator technologies could substantially increase this reconfigurability up to MHz or GHz speeds^31^.

## Discussion

A key concern for any optical system is the total optical loss. For many applications, the critical measure is power in the optical mode that is to be used for communication or sensing. For a 2D arrangement of grating couplers, this loss can be considered as the total power that will be diffracted into higher orders for generating beams or loss due to fill factor when sensing beams. For the suppression of all grating lobes, the separation between each optical IO is required to be less than one wavelength, λ. In our foundry-fabricated PIC, the minimum viable spacing was 49 μm, corresponding to about 32λ. The power loss from the main lobe due to grating lobes in a square grating coupler array was measured to be 15.4 dB^16^. We note that reducing the spacing would reduce this loss, but could increase the crosstalk between neighboring waveguides. The expected crosstalk for 750 nm spacing can be more than −10 dB from evanescent cross-coupling^46, 47^. This level of crosstalk would prevent closely packed phased-array PIC transceivers from functioning as low crosstalk mode multiplexers for communication applications. The use of an OMSI removes these constraints as the on-chip optical IO can be suitably separated to prevent this potential cross-coupling.

**Fig. 6:**
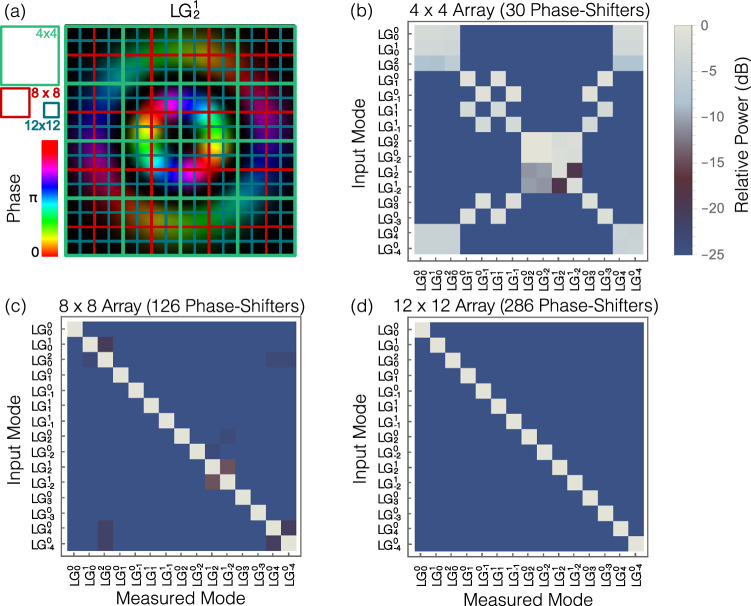
2D Nyquist sampling limitations. **a** Spatial information is commonly measured using a pixelated receiver. The recoverable information from this measurement approach is limited by the Nyquist sampling limits. If too few samples are recorded, high resolution information is lost and modes can not be uniquely resolved. We show the expected results for a pixelated receiver with **b** 4 × 4, **c** 8 × 8 and **d** 12 × 12 pixels decomposing 15 Laguerre Gaussian beams. For each case, we note the number of phase-shifters that would be required for each measurement.

Additionally, the number of grating couplers required for direct measurement is larger than that required for a system using an OMSI, as the number of couplers in the array does not satisfy the Nyquist sampling criterion for the system. Similar to time sampling, spatial sampling requires 2 sample points per spatial frequency in 1 dimension to achieve the Nyquist limit. For the direct illumination of a square grid of grating couplers, or a pixelated receiver, to resolve a waveform in 2D, the Nyquist sampling limit requires at least 4 sample points. Therefore, the use of an OMSI could result in more than 4 times fewer optical IOs, as we only need 1 sample point per mode. To illustrate this, the overlap integral is calculated between two sets of theoretical LG modes that vary in both azimuthal index *ℓ* and radial index *p*, with a beam waist of 4 mm for a different sampling resolution but the same effective aperture size of 20 mm. To achieve similar performance to our mode sorter, an 8-by-8 pixelated receiver is required, where *s*_mxt_ = −34.3 dB and *s*_cxt_ = −22.8 dB would be expected, as shown in Fig. 6. Comparatively, for mode sorting using a 7-by-7 pixelated receiver, *s*_mxt_ = −18.7 dB and *s*_cxt_ = −7.2 dB would be expected, which has more XT than the experimentally measured results from our proposed system with *s*_mxt_ = −22.1 dB and *s*_cxt_ = −10.1 dB. Therefore, an equivalent system without an OMSI would require 64 optical IO ports in a 2D arrangement, along with 126 phase-shifters (PS) for a binary tree arrangement, or 4032 for a fully connected mesh^39^. The OMSI achieves similar results with only 15 optical IO ports. The precise number required for a specific mode will change due to the geometric structure of the modes, but a square array or similar is required for arbitrary spatial mode decomposition.

Experimental losses in our feasibility study comprised a loss of 4.5 dB for each grating coupler, approximately 2 dB loss in the optical mesh. As our MPLC is implemented on an SLM, with approx. 90% reflectivity, and gold mirror within, with approx. 94% reflectivity, the folded path comprising 6 reflections from each leads to 8.5 dB due to absorptive losses. This resulted in an average loss of 19.5 dB, however, this is not fundamental and simply a function of the components selected for our study. We note that all of these losses can be substantially reduced using a fabricated OMSI and end-facet coupling. Additionally, end-facet coupling can substantially increase the bandwidth of optical frequencies usable in the system, as such approaches are commonly used within PIC-based wavelength multiplexers and demultiplexers. Experimental examples of MPLCs, using a similar folded optical path to our OMSI, have been demonstrated with an optical loss below 1 dB^45^. High efficiency edge couplers have been reported less than 1 dB. Therefore, this suggests that a packaged system could have a total loss of less than 4 dB with current commercially available fabrication processes, and our current experimental losses are not a limitation of the reported technique. Therefore, we project that our approach could provide an 11 dB gain compared to the state of the art 2D arrays of grating couplers used for beam forming that resulted in optical losses of 15.4 dB^16^.

In conclusion, we have presented a method for effective arbitrary modal sorting and generation, utilizing the combination of a PIC and an MPLC acting as an OMSI. The OMSI eliminates the issues that arise in optical phased-array antennas, such as grating lobes for generation and power loss due to low fill factor for measurement. Additionally, the OMSI can reduce the number of required optical IO by more than a factor of 4. This is potentially a huge advantage due to the complexities associated with electrical control and fabrication of large meshes of MZIs. Once packaged, our system could be widely used as a reconfigurable platform for a wider range of communication and sensing systems.

## Methods

### Experimental system

**Fig. 7:**
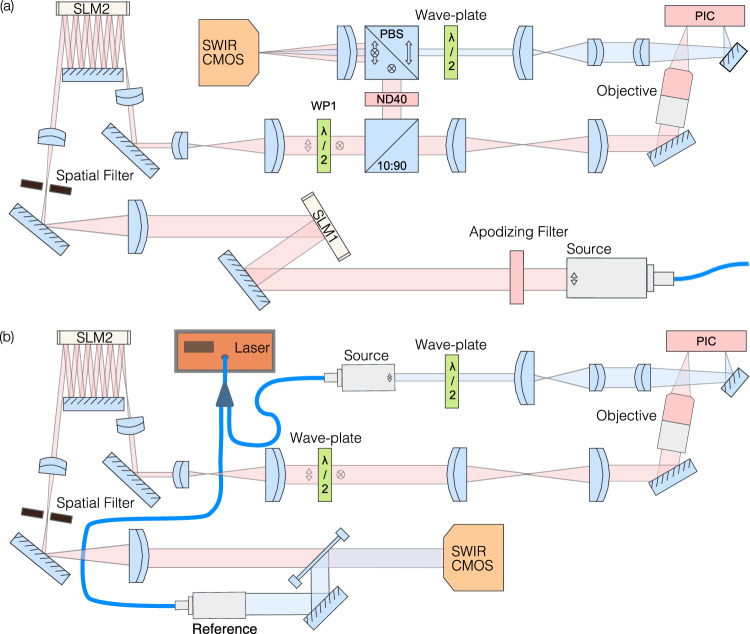
Experimental setup. **a** To experimentally measure the mode sorting performance, we generate a range of optical modes through the use of a Spatial Light Modulator (SLM). SLM1 is illuminated with a collimated laser source that is filtered by an apodizing filter to provide an even power distribution across its aperture. The first-order output from the hologram displayed on the SLM is spatially filtered and imaged onto SLM2, which displays the Multiple Plane Light Converter (MPLC). To record the output of the MPLC, we pick off 10% of the optical intensity and relay this to a short-wave infrared (SWIR) camera using a beam splitter. With image relay optics, 90% of the output power from the MPLC is imaged onto the PIC shown in Fig. 3. The output of the PIC is then relayed and imaged onto the same SWIR camera to support control of the phase shifters on the PIC. **b** Reversing the propagation of light through the system by coupling a collimated laser into the signal port on the PIC, optical modes can be generated. Using a fiber beam splitter, 10% of the source laser is collimated and interfered with the output of the MPLC to allow for off-axis digital holography to recover the phase and intensity of the generated structured beams.

**Fig. 8:**
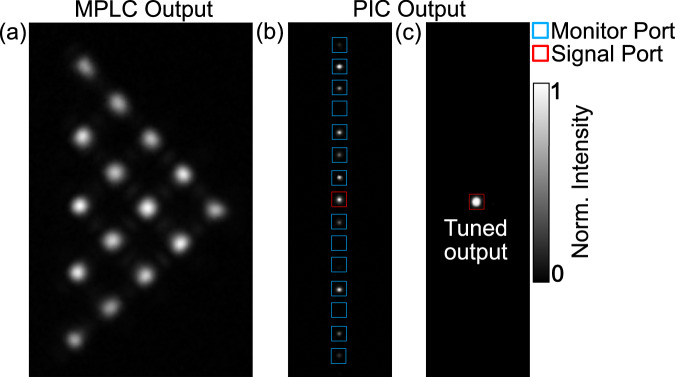
MPLC and PIC outputs. Short-wave infrared (SWIR) camerawas used to capture the output from the Multiple Plane Light Converter (MPLC) and is used to record the optical intensity from both the monitor ports and signal port. **a** An equally power-weighted superposition mode is input into the MPLC, showing intensity at each node in the triangular output. The presented images are normalized to the maximum intensity in each. **b** Without PIC tuning, various intensities can be measured at the monitor outputs, determined by the current set point of each phase shifter and subsequent interference between the input modes. **c** After applying the phase shifter tuning algorithm, all the power is superimposed and coupled out of the signal port.

Our experimental implementation utilizes FSO components to allow for prototyping and development; however, a packaged system could be readily made for future deployment for a range of applications. For system tuning and multiplexing studies, a phase-only SLM (Holoeye Pluto v1) is illuminated with the collimated output from a tunable C-band laser (Thorlabs TLX1) to generate optical modes, Fig. 7a. An apodizing filter is used to provide a uniform illumination across the surface of the SLM. Utilizing a computer-generated hologram with spatially controlled diffraction efficiency, the first-order beam is precisely shaped in phase and intensity to form the required spatial modes. These modes are then demagnified, such that a Gaussian beam has a mode field diameter of 500 μm, as required to match the optimized design parameters of the OMSI. The OMSI is patterned on a second-phase-only SLM (Holoeye Pluto v2.1), where a folded optical path allows for the sequential illumination of masks I–VI. To monitor the alignment of the OMSI, a 10:90 beam splitter (Thorlabs BS044) is used to image the optical field on a short-wave infrared (SWIR) camera (Raptor OWL 640M), see Fig. 8. As our feasibility study is conducted using an SLM, a further multi-stage de-magnification is required for the illumination of the PIC input couplers.

The SLM was used to allow for prototyping and optimization of the phase masks to match the fabricated optical mesh. Once optimized, the masks are not altered as we change the PIC settings of the OMSI for different spatial mode-sets used in our experiment. Therefore, the OMSI could in future be replaced with a fabricated passive optical element. Multi-plane transformers for different mode types have been previously demonstrated with losses less than 1 dB^22^ and sorting of over 1000 spatial modes^48^. This indicates that this design approach can be scalable when fully packaged in an optical system meant for efficient coupling into and out of integrated photonic chips. Our implementation required 6 transformation planes to efficiently multiplex 15 spatial modes, however, scaling to more modes may require additional planes, leading to additional reflective losses and subsequently lower optical efficiency.

### Channel capacity

We estimated channel capacity for our system by calculating the function

2 $$c=m\,b\,{{\mbox{Log}}}_{2}\left(1+\frac{{\sum }_{i=1}^{m}{P}_{ii}}{m{{\mbox{n}}}_{{{\rm{e}}}}+{\sum }_{i=1}^{m}{\sum }_{j=1}^{m}{P}_{ij}{\mbox{if}}\,i\, \ne\, j}\right)$$

where *m* is the number of spatial modes, *b* is the bandwidth in Hz, and *n*_e_ is an assumed electrical noise equivalent to a single channel SNR of 27 dB. Equation (2) is a modification of the Hartley–Shannon theorem incorporating the additional bandwidth available from spatial channels in addition to wavelength^49^. The value of 27 dB was chosen to be the middle of the SNR range of 20–35 dB that is common for diode-based multi-GHz-speed receivers^50^.

### Crosstalk calculations

To analyze our data we determine XT in two forms, the mean XT, which we represent as the variable *s*_mxt_, and cumulative XT, *s*_cxt_. *s*_mxt_ is deduced by determining the mean value of the power in all the off-diagonal components as ratio of the measured power, *P*(*i*, *j*) in that element of the 2D array to the maximum power in any diagonal element. This can be mathematically calculated by the function

3 $${s}_{{{\rm{mxt}}}}=10\,{{\mbox{Log}}}_{10}\left(\frac{1}{{m}^{2}-m}{\sum }_{i=1}^{m}{\sum }_{j=1}^{m}\frac{{P}_{ij}}{\,{\mbox{max}}(P)}{\mbox{if}}\,i\,\ne \,j\right).$$

The cumulative XT, *s*_cxt_, is calculated for all 15 modes by determining the ratio of total power along the diagonal and the total power in all off-diagonal elements. This is calculated by the function

4 $${s}_{{{\rm{cxt}}}}=10\,{{\mbox{Log}}}_{10}\left(\frac{{\sum }_{i=1}^{m}{\sum }_{j=1}^{m}{P}_{ij}\,{\mbox{if}}i\,\ne \,j}{{\sum }_{i=1}^{m}{P}_{ij}{{\mbox{where}}}\,j=i}\right).$$

### LG overlap integral

For the simulation of mode-sorting with a pixelated receiver, we calculate, explicitly, the complex profile of the LG mode, using the function in Cartesian coordinates. In the waist plane of an LG mode the complex amplitude is given by

5 $$\begin{array}{rcl}{\Psi }_{\ell ,p}(x,y) & = & {C}_{\ell ,p}{\left(\frac{\sqrt{2\left({x}^{2}+{y}^{2}\right)}}{{w}_{0}}\right)}^{| \ell | }{L}_{p}^{| \ell | }\left(\frac{2\left({x}^{2}+{y}^{2}\right)}{{w}_{0}^{2}}\right)\\ & \times & \exp \left(\frac{-\left({x}^{2}+{y}^{2}\right)}{{w}_{0}^{2}}\right)\exp \left[i\ell\, {\tan }^{-1}\left(\frac{y}{x}\right)\right],\end{array}$$

where *w*_0_ is the waist size, ${L}_{p}^{| \ell | }(x)$ is the Laguerre polynomial for the azimuthal, *ℓ*, and radial, *p*, mode indices, and *C*_*ℓp*_ is the amplitude normalization term^51, 52^, given by

6 $${C}_{\ell ,p}=\frac{1}{{w}_{0}}\sqrt{\frac{2p!}{\pi \left(p+| \ell | \right)!}}.$$

We compute $\exp \left[i\ell {\tan }^{-1}\left(\frac{y}{x}\right)\right]$ through the use of an arctan function that accounts for which quadrant a point (*x*, *y*) is located in. As LG modes form a complete orthonormal basis set, they can be used to express any beam cross-section *ψ*(*x*, *y*) as a superposition of LG modes:

7 $$\left|\psi \right\rangle ={\sum }_{\ell =-\infty }^{\infty }{\sum }_{p=0}^{\infty }{A}_{p}^{\ell }\left|{\Psi }_{l,p}\right\rangle $$

where the contribution of each mode is given by

8 $${A}_{p}^{\ell }=\langle {\Psi }_{l,p}| \psi \rangle ,$$

and the relative power in each mode is simply

9 $${P}_{\ell ,p}=| {A}_{p}^{\ell }{| }^{2}=| \langle {\Psi }_{l,p}| \psi \rangle {| }^{2}.$$

The Cartesian coordinates *x* and *y* are varied such *x* ≤ *n*_*p*_, *y* ≤ *n*_*p*_, and the matrix is calculated for a set of modes equivalent to those used in our experimental measurements, with *n*_*p*_ set to 4, 8, and 12, respectively.

### Inclusion and ethics

During experimental design, development, and execution, we consider the ethical standards in relation to the technology utilized in this research work. Our data does not include personal or potentially socially biased data. All efforts were taken to be inclusive of local researchers during this research project.

## Data Availability

Data underlying the results presented in this paper are not publicly available at this time but may be obtained from the authors upon request.

## Code availability

Computer code used for processing the results presented in this paper is not publicly available at this time but may be obtained from the authors upon request.
